# Motometrics: A Toolbox for Annotation and Efficient Analysis of Motor Evoked Potentials

**DOI:** 10.3389/fninf.2019.00008

**Published:** 2019-03-26

**Authors:** Shivakeshavan Ratnadurai Giridharan, Disha Gupta, Ajay Pal, Asht M. Mishra, N. Jeremy Hill, Jason B. Carmel

**Affiliations:** ^1^Motor Recovery Lab, Burke Neurological Institute, White Plains, NY, United States; ^2^Department of Neurology, New York University School of Medicine, New York, NY, United States; ^3^Comprehensive Epilepsy Center, NYU Langone Health, New York, NY, United States; ^4^Brain Mind Research Inst., Weill Cornell Medicine Medical College, New York, NY, United States

**Keywords:** motor, evoked, MEP, EMG, recruitment, analysis, software

## Abstract

Stimulating the nervous system and measuring muscle response offers a unique opportunity to interrogate motor system function. Often, this is performed by stimulating motor cortex and recording muscle activity with electromyography; the evoked response is called the motor evoked potential (MEP). To understand system dynamics, MEPs are typically recorded through a range of motor cortex stimulation intensities. The MEPs increase with increasing stimulation intensities, and these typically produce a sigmoidal response curve. Analysis of MEPs is often complex and analysis of response curves is time-consuming. We created an MEP analysis software, called Motometrics, to facilitate analysis of MEPs and response curves. The goal is to combine robust signal processing algorithms with a simple user interface. Motometrics first enables the user to annotate data files acquired from the recording system so that the responses can be extracted and labeled with the correct subject and experimental condition. The software enables quick visual representations of entire datasets, to ensure uniform quality of the signal. It then enables the user to choose a variety of response curve analyses and to perform near real time quantification of the MEPs for quick feedback during experimental procedures. This is a modular open source tool that is compatible with several popular electrophysiological systems. Initial use indicates that Motometrics enables rapid, robust, and intuitive analysis of MEP response curves by neuroscientists without programming or signal processing expertise.

## Introduction

To assay the descending motor system, muscle responses to motor cortex stimulation are measured as the motor evoked potential (MEP) using electromyography (EMG). MEPs are used to measure changes in the motor system, due to neurological diseases or injury (Nitsche and Paulus, 2000; Buccino et al., 2005), and to measure the effects of therapy (Liepert et al., 1998; Sindhurakar et al., 2017). A characteristic of MEPs is that as stimulation strength is gradually increased, the MEP increases exponentially, then exhibits a linear relationship, and finally begins to saturate as stimulation intensity is increased (Devanne et al., 1997; Boroojerdi et al., 2001; Luft et al., 2001). This gives an “S” or sigmoid curve to the plot of stimulus intensity vs. MEP. This characteristic curve is known as the *recruitment curve* as it describes indirectly, the MEP obtained by “recruiting” motor units (Fuglevand et al., 1993).

**Figure 1:**
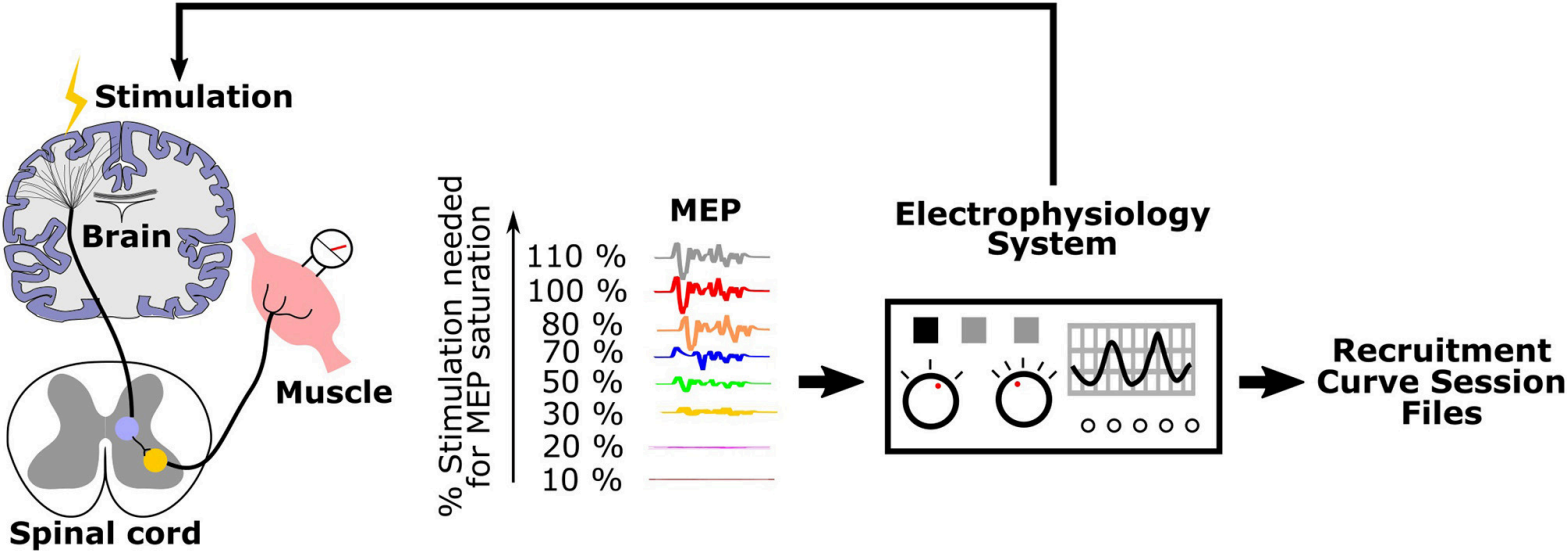
Generation of recruitment curves during an experimental session: Stimulation is applied to the motor cortex with varying intensities. Corresponding MEPs are recorded and saved as recruitment curve session files by the electrophysiology system.

The process for generating recruitment curves is illustrated in Figure 1. Stimulation of motor cortex is done with electrodes or magnetic stimulation, and EMG electrodes are placed in or on top of the muscle(s) of interest. An electrophysiology system is used both for stimulation and for recording MEPs. The motor cortex is stimulated with intensities ranging from eliciting no MEP (sub-threshold) to saturation, when the magnitude of MEPs do not increase with increasing stimulation intensity. The electrophysiology system records and saves these signals as files that we term “recruitment curve session files.”

**Figure 2:**
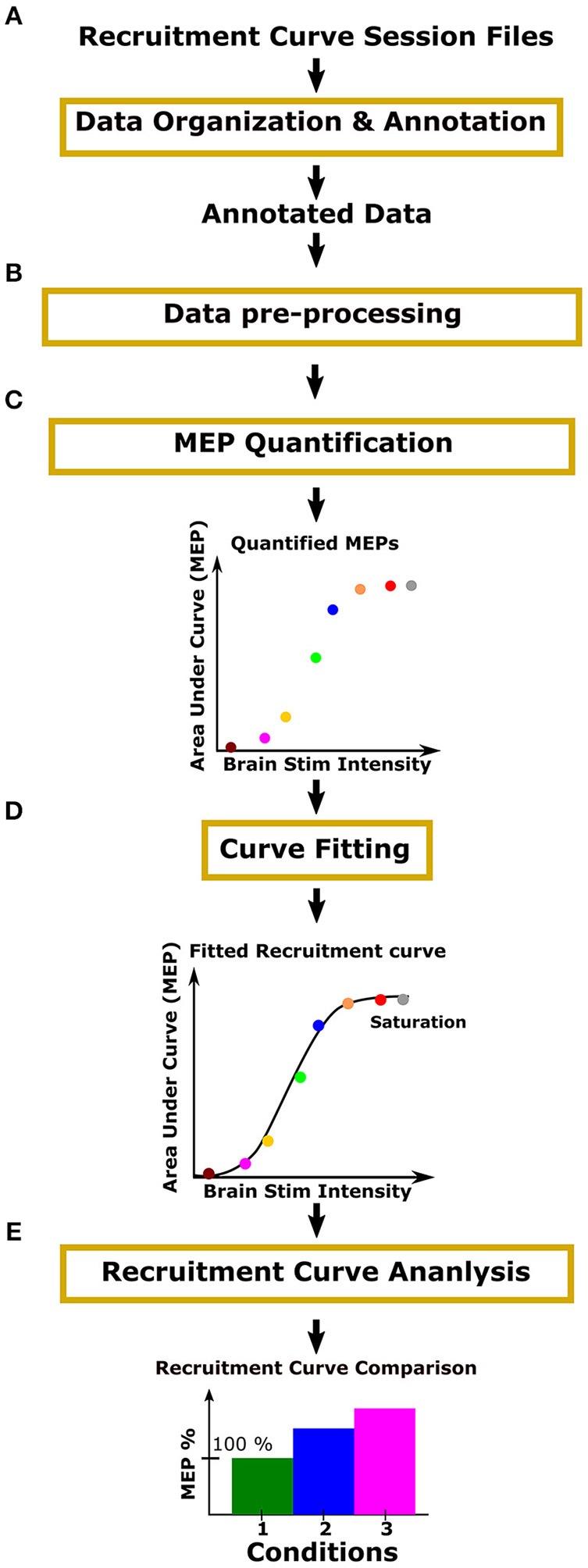
Process overview**:** Motometrics uses modules to organize experimental data, quantify MEPs, fit recruitment curves, and compare recruitment curves across conditions. **(A)** The user organizes and annotates MEP data files relevant for the generation of recruitment curves. **(B)** The user inspects the quality of data and removes data with artifacts. The user may also select to filter the data to remove noise and correct baseline drift. **(C)** The user then chooses a metric, such as area under the curve or peak amplitude, to quantify the organized MEP data. **(D)** Recruitment curves are generated using a curve fitting algorithm. **(E)** The user next selects a recruitment curve metric (e.g., slope) to quantify and compare across curves.

The process for analyzing recruitment curves is shown in Figure 2. For the purpose of analysis, one must annotate recruitment curve session files with information such as the range of stimulation intensities used and the number of repeated MEP recordings made for each stimulus intensity (Figure 2A: Data organization and annotation). The next step is to preprocess the data in order to specify the relevant MEP signal segment, and to screen for outliers and artifacts (Figure 2B: Data pre-processing). Using this annotated and preprocessed data, one must then apply signal processing methods such as MEP rectification and computing area under the curve to quantify the MEP signals across a range of intensities (Figure 2C: MEP quantification). Following this, one must fit an appropriate curve through quantified MEPs to obtain a recruitment curve (Figure 2D: Curve fitting). Finally, recruitment curves may be analyzed and compared (Figure 2E: Recruitment curve analysis).

In Figure 2, the yellow outline boxes represent tasks that the experimentalist would need to perform in order to go from acquiring MEP signals, to constructing recruitment curves and analyzing them. These tasks can be very time consuming and often the analysis is only performed offline after experimentation. These tasks also often require high levels of technical expertise that experimental neuroscientists may or may not have. Additionally, without a standard software, many researchers may be re-inventing similar processes for recruitment curve fitting. To our knowledge, there does not exist a software especially for recruitment curve fitting and analysis. There are some software that focuses only on MEP quantification, but even those are either electrophysiology hardware system dependent, or they are specific to the type of stimulation and EMG electrodes used (MEPHunter, a Free Software for Signal Visualization and Analysis, 2014).

The ideal MEP recruitment curve analysis software will satisfy the requirements of neuroscientists with varying degrees of technical expertise, while providing an easy-to-use graphical user interface (GUI) for quick analysis. Specifically, it will be independent of the acquisition hardware, the mode of stimulation (TMS, electrical, or optogenetic etc.), and type of EMG electrode (surface vs. indwelling) used. The user interface must be intuitive to use and the software itself should be fast enough to process hundreds of MEPs within a few seconds. Working toward this ideal solution, we built Motometrics—an open source, multi-platform MATLAB software.

Motometrics enables an experimentalist to quantify MEPs and fit and analyze recruitment curves with minimal technical expertise. The software fills in default values, with the assistance of a GUI wizard, where appropriate, and displays pop-up messages to highlight errors with suggestions about how to correct them. Motometrics consists of five primary modules as illustrated in Figure 2. The modules perform the necessary functions for annotating and organizing MEP data, preprocessing and screening MEPs, quantifying MEPs, fitting recruitment curves to quantified MEPs, and finally analyzing and comparing multiple recruitment curves. Additionally, Motometrics introduces a common MEP data storage format in MATLAB. By converting exported data from different electrophysiology systems to this common format, hardware independent analyses can be performed and data can be shared between different research groups. In the following methods section we discuss the functions of each of the five Motometrics modules in detail.

## Methods

This section is organized by the five critical components that make up Motometrics as outlined in Figure 2. We first introduce the organization of data for Motometrics. Next, we describe how relevant data is extracted from the entire file and how preprocessing helps to identify outliers. We will then describe the process by which the organized MEP data can be used to generate recruitment curves. We also present the methods by which we compare recruitment curves obtained from different experimental conditions. We finally describe how Motometrics can satisfy the analytical requisites of both the non-technical and advanced user.

Motometrics is currently in the beta stage (version 1.2). Motometrics was built using an iterative development model. We began by focusing on a core set of functionalities and features and based on analysis of user feedback, modified the code as needed. It has also been tested by multiple labs from other institutions. Motometrics is hosted as an open repository that allows contributions from the public. A wiki page has been set up to guide new users as well. As the primary developers, we welcome contributions in the form of bug fixes, code enhancements or suggestions. The open source Motometrics software may be downloaded from an online repository: https://bitbucket.org/burkemedicalresearch/motometrics/downloads/?tab=tags.

### Data Organization and Annotation

**Figure 3:**
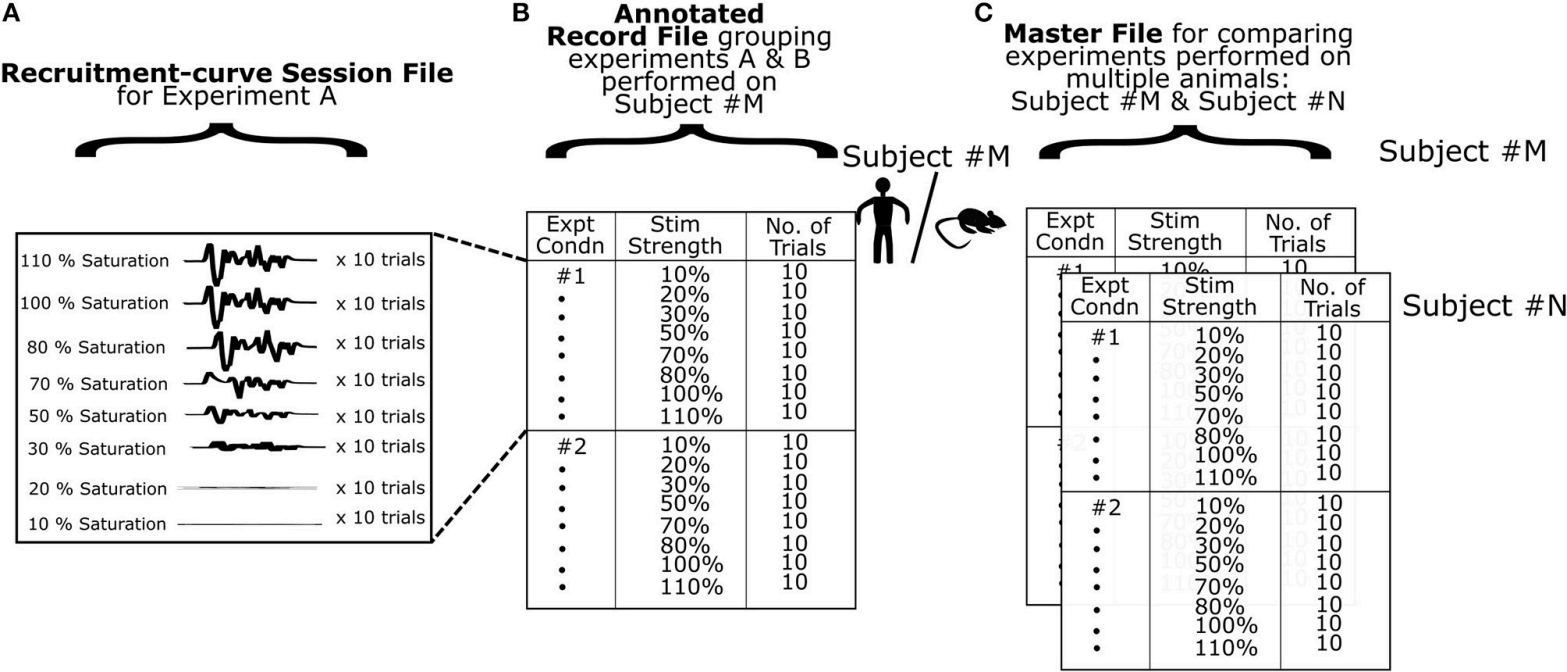
Organization of data. **(A)** A single experimental recording consists of MEPs obtained using a defined range of stimulation intensities. At every stimulus intensity multiple trials are obtained. The data is saved as a multidimensional matrix in a MATLAB data file. **(B)** At the next level of data organization, we specify multiple experimental recordings in a record file for a single subject. Here, we utilize experiment labels to denote different experimental conditions for that subject. The software can use a record file to generate and analyze recruitment curves for a single subject. **(C)** Finally we use a master file to group multiple subjects or data from the same subject across time with similar experimental conditions for comparison of recruitment curves. The software can use a master file to enable detailed comparisons across multiple subjects.

MEP data used in this software comes from files created by the electrophysiology hardware/software system after stimulation through a range of values. We term these “raw recruitment-curve data files” (Figure 3A). Each of these files contain all relevant MEP data required to generate a complete recruitment curve in a single session under a fixed experimental condition. To minimize variability across recordings (Kiers et al., 1993), it is common to stimulate and measure MEPs multiple times for each stimulation intensity. A single stimulation and its corresponding MEP is termed a “trial”. Typically, 20 or more trials are recommended per stimulation intensity for humans (Biabani et al., 2018), and 10–20 trials are recommended per stimulation intensity for rodents (Mishra et al., 2017; Garcia-Sandoval et al., 2018). In practice however, the exact number of trials may depend on various factors of the study and the exact number of trials must be chosen by the investigators based on the hypotheses and study population. Motometrics imports these raw recruitment-curve data files to quantify MEPs and generate recruitment curves. However, these files must first be reformatted and sufficiently annotated to provide all necessary information to Motometrics. This annotation is required because recruitment-curve session files only provide raw MEP signals without information on the stimulus intensities used or the number of trials taken for each stimulus intensity.

A GUI for annotation is provided by Motometrics to enable the user to specify recruitment-curve session files along with the stimulus intensity and trial information. This annotated information for each subject is saved into a new file created by Motometrics: the “record” file. Furthermore, for the sake of comparing multiple recruitment-curves, a single record file may contain annotation of more than one recruitment-curve session files, as illustrated in Figure 3B.

When the user wishes to quantify MEPs and analyze recruitment curves across multiple subjects, Motometrics provides a way to group multiple record files into a single master file as shown in Figure 3C. In the following subsections, we elaborate on the structure and organization of recruitment-curve session files, record files, and the master file. We also provide examples of these MATLAB files in the Supplementary Section of this paper.

Although the primary focus of Motometrics is to generate and analyze recruitment curves, it is also possible to analyze data that are not part of a recruitment curve. In this case, mean MEP magnitudes are compared directly across conditions.

#### Structure of Recruitment-Curve Session Files

The data file formats of recordings vary across different electrophysiology systems. While most electrophysiology systems do provide the option to convert or export recorded data to a MATLAB format, the structure of the data itself can vary a lot. Before the user can begin to use Motometrics to analyze MEP data recordings, the user must first convert recruitment-curve session files into a MATLAB file that is compatible with Motometrics. We provide a sample recruitment-curve session file in the Supplementary Section as an example. The original data is obtained through a CED system (CED Micro 1401, Cambridge Electronic Design Ltd., Cambridge, UK) using Signal software, version 5.08.

To generate a recruitment curve, the stimulation intensity must be varied across a range of values. Specifically, for the recruitment curve to be valid, stimulation must begin below the threshold for inducing an MEP, and reach saturation (increasing stimulation intensity does not increase the MEP). At each stimulation intensity, multiple MEP recordings (trials) are typically taken to reduce variability. So, for example, if 10 trials are recorded per stimulation intensity, then for a range of 6 stimulation intensities, we would have a total of 60 MEPs. The dimensions of the file structure so far can be described as: [MEP signal], [stim intensity × trials[stim intensity]] as illustrated in Figure 3A. Here, trials[stim intensity] indicates the number of trials for each stimulation intensity. The number of trials can be different for each intensity. Additionally, an experimentalist might wish to record MEP from multiple muscles and then perform analyses on data corresponding to each muscle, respectively. For this purpose, Motometrics can analyze MEPs from multiple muscles using an additional dimension to the data: channels, where each channel represents a recording from a different muscle/electrode. This causes the data to take shape with the following dimensions:

$$\begin{array}{l} \left[\text{MEP signal length }\right],\;\left[\text{channels}\right],\;\left[\text{stim intensity }\times \text{ trials}\right] \end{array}$$

Motometrics assumes that the MEP signals are organized in ascending order of stimulation intensities. If there is a need to randomize across trials and stimulation intensities for MEPs, then the data must be reshuffled to the correct order of ascending stimulation intensities when saving as a recruitment curve session file. We have provided example scripts in the Motometrics repository that demonstrate how a user may reorder the MEP data when creating recruitment curve session files.

#### Organization of Record Files

A record file contains one or more recruitment curve sessions for a single subject. Within record files, we construct a table to describe the experimental session(s) as shown in Figure 3B. In the first column, we provide a unique number as a label for each experimental session. The second and third columns list the stimulation intensities and number of trials done for each intensity. The *Record Manager* tool provided in our software enables the user to perform this task. The first recruitment curve session of every record is always assumed to be the baseline (normal state/control state) condition against which other sessions are to be compared. If baseline session information is recorded later in the experiment, then when creating and annotating the record file, this baseline session must be specified as the first recruitment curve session in the record file.

#### Organization of Master Files

To analyze recruitment curves across groups of subjects we compile record files of different subjects into a single master file as shown in Figure 3C. A prerequisite for this is that the number of experimental sessions performed per subject is the same across the group. i.e., the number of recruitment curve sessions in each record file should be the same, although the total number of trials or stimulus intensity ranges may vary within and across record files. The user may utilize the *Master File Manager* tool to create a master file and add record files under it. In the Results, we will demonstrate how data organization helps us perform analyses.

### Data Pre-processing

Preprocessing helps to define the epoch that contains the MEP and identify trials with artifacts. Artifact removal helps subsequent phases of the software pipeline to reliably process the MEPs and provide meaningful results. Motometrics preprocessing of data is performed in three steps as detailed in the following subsections.

#### Filtering the Signal

A band pass filter may be applied to correct baseline drift or wire movement artifact. The filter is a band pass Butterworth filter (Selesnick and Burrus, 1998) applied with a zero-phase filtering approach to prevent phase distortion. The lower cutoff frequency and upper cutoff frequency is specified by the user. Currently default values of 5 Hz and 600 Hz are used, respectively. The filter order was estimated through MATLAB with an acceptable passband ripple of 4dB, and a stopband attenuation of 30dB. The advantage of the Butterworth filter is that its frequency response is maximally flat in the passband, i.e., the power of all frequencies in the passband are almost equally affected by the filter. As in the case of epoch and channel selection above, these preprocessing parameters can either be applied to all record files automatically, or may be applied with different pre-processing parameters to individual record files for finer control of data.

An optional notch filter for removing power line noise and associated harmonics from recorded data is also available in Motometrics. The user may choose to enable it and select the number of harmonic levels to remove (up to 6 multiples of 60 Hz).

#### Selecting the Channel and Epoch Containing the MEP

The next step involves specifying the time window that contains the MEP with respect to the stimulation onset. This window is fixed for all experiments within a single record file based on the assumption that the duration of MEPs are approximately the same. Then, the user specifies the recording channel that corresponds to the EMG electrodes of interest. These data selection parameter settings can either be applied to all record files automatically, or may be applied with different pre-processing parameters to individual record files for finer control of data analysis.

#### Artifact Removal

The final step of pre-processing enables artifact rejection and occurs after a master file or record file is loaded. While currently there is no automated artifact rejection module in Motometrics, data visualization tools enable efficient selection and removal of unwanted MEP data. Here the entire dataset in a record file may be reviewed directly as MEPs or as a heat map of all trials (as shown in Results section Data Selection and Pre-processing). An example is shown in Figure 8. This enables one to visually scan for noisy trials and artifacts due to motion, electrical interference, or bad contact. The trial can be visualized and removed if it is found to be noise/artifact.

### MEP Quantification

After data has been annotated and preprocessed, the MEPs have to be quantified in order to generate a recruitment curve. Quantification is applied to every MEP trial in a recruitment curve session file, and the mean values across all trials for each stimulation intensity are obtained. There are 4 different ways of quantifying MEP signals.

#### Area Under the Curve

The *area under the curve* (AUC): To find the AUC, we first rectify the signal by taking the absolute value of the MEP. The AUC is quantified as:

$$\begin{array}{l} \int_{a}^{b}f\left(t\right)dt=\frac{b-a}{2N}\overset{N}{\underset{n=1}{\sum }}\left[\;f\left(t_{n}\right)+f\left(t_{n+1}\right)\right] \end{array}$$

Where *f(t)* represents the rectified MEP at time point *t*, [a,b] specify the starting and ending points of the MEP, and N is the number of uniformly sampled points between [a,b]. The area is calculated using the trapezoidal rule and is achieved using the “trapz” function in MATLAB.

#### Root Mean Square

Another similar available metric is the *root mean square* (RMS) metric. This measures the average amplitude of a signal over its duration. It is approximately equal to the AUC divided by the duration of the signal.

$$\begin{array}{l} f_{rms}=\;\sqrt{\frac{1}{T_{2}-T_{1}}\int_{T_{1}}^{T_{2}}{\left[\;f\left(t\right)\right]}^{2}dt} \end{array}$$

Here, f_rms_ is the root mean squared value of the rectified MEP signal *f(t)* measured between time T1 and T2.

#### Peak to Peak

The peak-to-peak amplitude is the difference between the maximum and minimum values of the MEP.

#### Latency

While the three metrics described previously measure different aspects of the magnitude of the MEP signal, the final metric available in Motometrics measures the delay between stimulation and MEP. The *latency metric* is calculated as the time from the end of stimulation to when the signal achieves a certain percentage above baseline (provided by the user) of the absolute signal's maximum. The user can access MEP latency data either as a latency vs. stimulus intensity plot in Motometrics, or by saving quantified MEP data. Recruitment curves cannot be fit to latency data since they do not follow a sigmoidal stimulus-response profile.

### Recruitment Curve Fitting

We fit a recruitment curve to the experimental data points corresponding to quantified MEPs. We use a logistic function, a two-state condition of the Boltzmann function, that may be characterized by 4 parameters. The lower asymptote (P), the upper asymptote (M), the slope of the sigmoidal curve (L), and the center of the sigmoid curve (K). The parameters when used in the following equation, completely describe a sigmoidal curve.

$$\begin{array}{l} y\left(x\right)=P+\;\frac{M-P}{1+Qe^{{-e}^{L}\left(x-K\right)}} \end{array}$$

The goal for recruitment curve fitting, is to determine the parameters *P, M, L*, and *K* such that the corresponding sigmoid curve provides the best fit to the data points while minimizing sensitivity to outliers (Wichmann and Hill, 2001). In the case of our data, the *x* values of the data points represent stimulation intensities and y values represent MEP magnitude using a selected signal metric (See Methods section *MEP Quantification*). The initial estimate for sigmoidal curve parameters: *P, M, L* and *K* can be critical for the convergence to a good fit. The parameter *Q* is set to the initial value of *y(0)*. Our approach to estimate our initial values is as follows:

- Lower asymptote (*P*) calculated as median of 5 values of *y* centered on the 5th percentile.
- Upper asymptote (*M*) calculated as median of 5 values of *y* centered on the 95th% percentile.
- Logarithmic slope (*L*) calculated as tangent to the curve.
- Midpoint (*K*) calculated as the median of 2 *x*-values from the center, Where the center is determined as: abs(*y* − (P+M)/2).

Starting with these initial estimates of the parameters we use the non-linear Nelder-Mead local optimization method (Lagarias et al., 1998) to identify the final parameter values that provide the best fit (minimizing least square error) to the data points. This optimization method runs iteratively until the error falls below a user specified value (default of 0.1). In the case where this does not happen, fitting ends if the change in fit error is below a certain threshold (0.01) or if the fit does not improve over 10 consecutive iterations. This approach for estimating initial parameters, as well as the non-linear optimization method for identifying the optimal parameters, was empirically observed to prioritize resistance to outliers over simply minimizing the least squares fit.

The goodness of fit between the estimated sigmoidal curve and the actual data is represented by the coefficient of determination (R^2^). The R^2^ coefficient ranges from 0 to 1, where 1 indicates a perfect fit to the data. Additionally, after the recruitment curve is fitted, a check for saturation is done. This is performed by considering the last three points of the curve, fitting a linear segment through these points, and verifying if the slope of this line is <0.2 (by empirical observation). A slope of zero indicates that the last three quantified MEPs are approximately in the same range (indicating saturation). If the slope fails to satisfy this condition, the user is alerted through a warning when viewing the S-curves through the data explorer as described in Results section *MEP quantification and Generation of recruitment curves*. The warning only alerts the user to this issue but does not stop them for proceeding with further analysis. It is left up to the discretion of the user on how to proceed.

### Recruitment Curve Analysis

A recruitment curve is characterized by its sigmoidal shape. The initial rise in the curve indicates the early recruitment of motor units, typically innervated by smaller motoneurons (Belanger and McComas, 1981). The slope of the linear portion of the curve represents the rate at which motor units get recruited as stimulation intensity is increased (Fuglevand et al., 1993). The saturation of the curve for higher stimulation intensities, suggests that recruitment of motor units has reached its maximum potential and there are few motor units left to contribute to an MEP. Motometrics uses three different metrics (next 3 sections) to quantify the recruitment curve. These metrics enable comparison of multiple recruitment curves that can reveal differences in motor unit recruitment. These metrics can be applied to a single subject for the purpose of comparing the effects of different experimental conditions as well as the effects of different experimental conditions across a range of subjects.

#### MEP Metric

**Figure 4:**
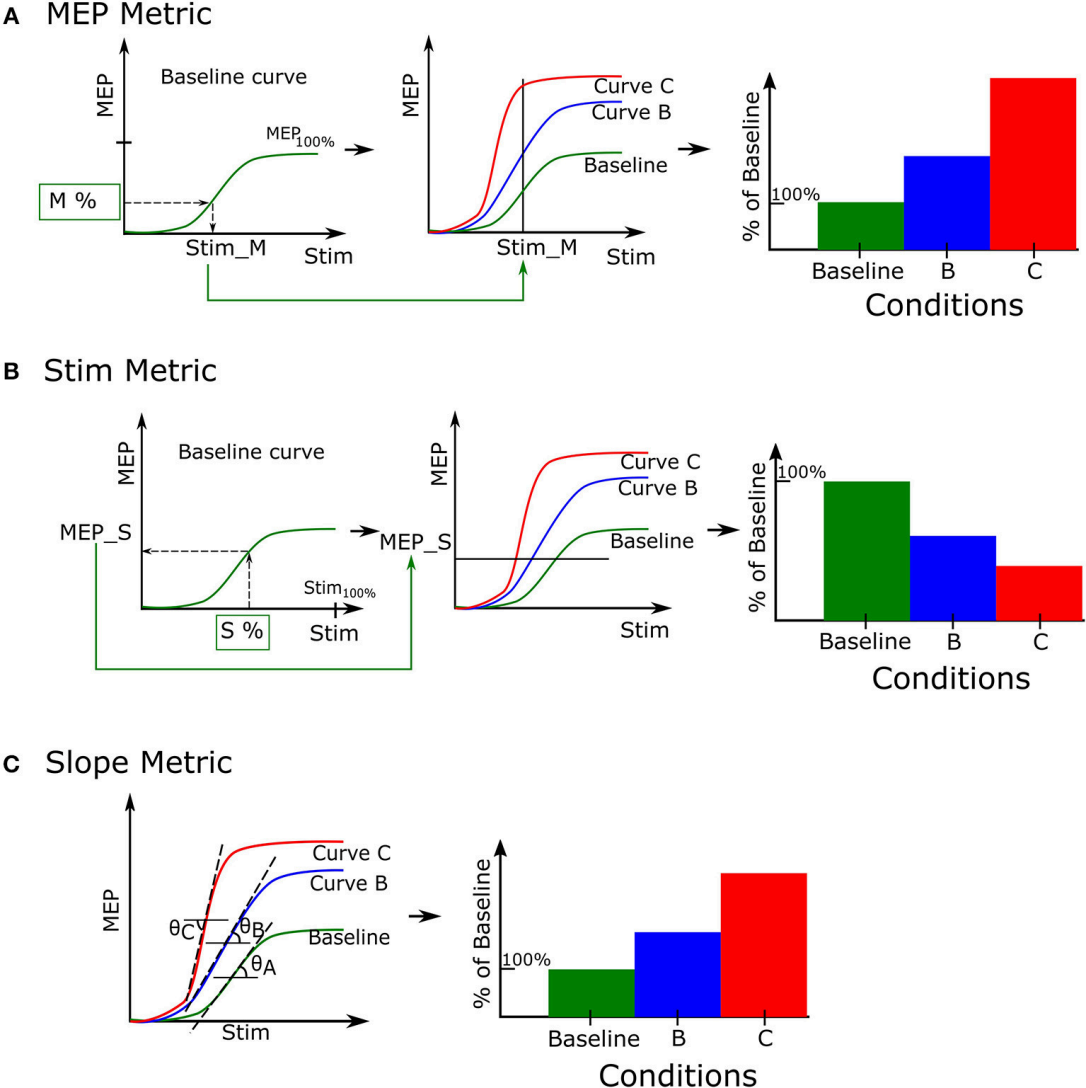
Recruitment curve metrics. A set of recruitment curves from a single record file may be evaluated using 3 different metrics. **(A)** The MEP metric enables recruitment curves to compare their quantified MEP responses for a fixed stimulation. **(B)** The Stimulation metric compares stimulation intensities required to elicit a certain value of the quantified MEP. **(C)** The Slope metric estimates the tangent of the angle made by the linear portion of the recruitment curve with the horizontal line.

The *MEP metric* identifies the MEP magnitudes for a given stimulation intensity. This can be conceptualized as a vertical cut on the sigmoidal curves. In Figure 4A we illustrate how this metric is applied to three recruitment curves for the purpose of comparison. We first designate one of the recruitment curves to be a baseline reference (usually the first recruitment curve in a set). For a given percentage of baseline MEP, *M* %, we identify the corresponding stimulation intensity (*Stim_M*) that can elicit this MEP response on the baseline recruitment curve (Figure 4A, left). Next, we determine the MEP magnitudes for the same stimulus intensity, *Stim_M* (Figure 4A, center). Finally, these MEP magnitudes are expressed as a percentage change from the baseline MEP response and can be represented, for example, as a bar graph (Figure 4A, right).

#### Stimulation Metric

We quantify the *stimulation metric* as the stimulation intensities required to elicit the same MEP magnitude across conditions. This can be conceptualized as cutting the curves with a horizontal line. To do this, we first select a percentage of maximum stimulation intensity, *S* %, and identify the corresponding MEP (*MEP_S*) it produces in the baseline recruitment curve (Figure 4B, left). We use this MEP value to identify which stimulation intensities can generate the same MEP magnitude in recruitment curves B and C, respectively (Figure 4B, center). Finally we normalize these identified stimulation intensities to the baseline (Figure 4B, right).

#### Slope Metric

The third measurement of the recruitment curve is the *slope metric*. This measures the slope or gain of the linear portion of the recruitment curve, as shown in Figure 4C, left. The metric quantifies the rate at which motor units are recruited as stimulation intensity is increased. This is done by calculating the derivative of the recruitment curve and identifying portions where the derivative is constant and nonzero. We then perform simple linear regression through this portion to calculate the slope. Finally the slope is either normalized by the baseline recruitment curve slope to generate comparison data for analysis (see Figure 4C right), or the raw slopes may be directly plotted based on user preference. A higher value indicates a steeper slope than the baseline curve.

### Motometrics for the Non-technical and Advanced User

Motometrics has been designed to serve the analytical needs for both the non-technical and advanced user. Introducing the parameters that configure Motometrics upfront, offers the new or non-technical user complete control and understanding over the analytical processes as outlined in Figure 2. Data transparency on the other hand, provides the advanced user with access to data corresponding to each stage in the processing pipeline (Figure 2). This data may be used for visualization purposes or for custom analyses. The first of the following subsections describes the way Motometrics assists with selection of parameters for the new or non-technical user. The second subsection describes how data transparency is implemented in Motometrics for advanced users.

#### Motometrics Parameter Wizard for New or Non-technical Users

**Figure 5:**
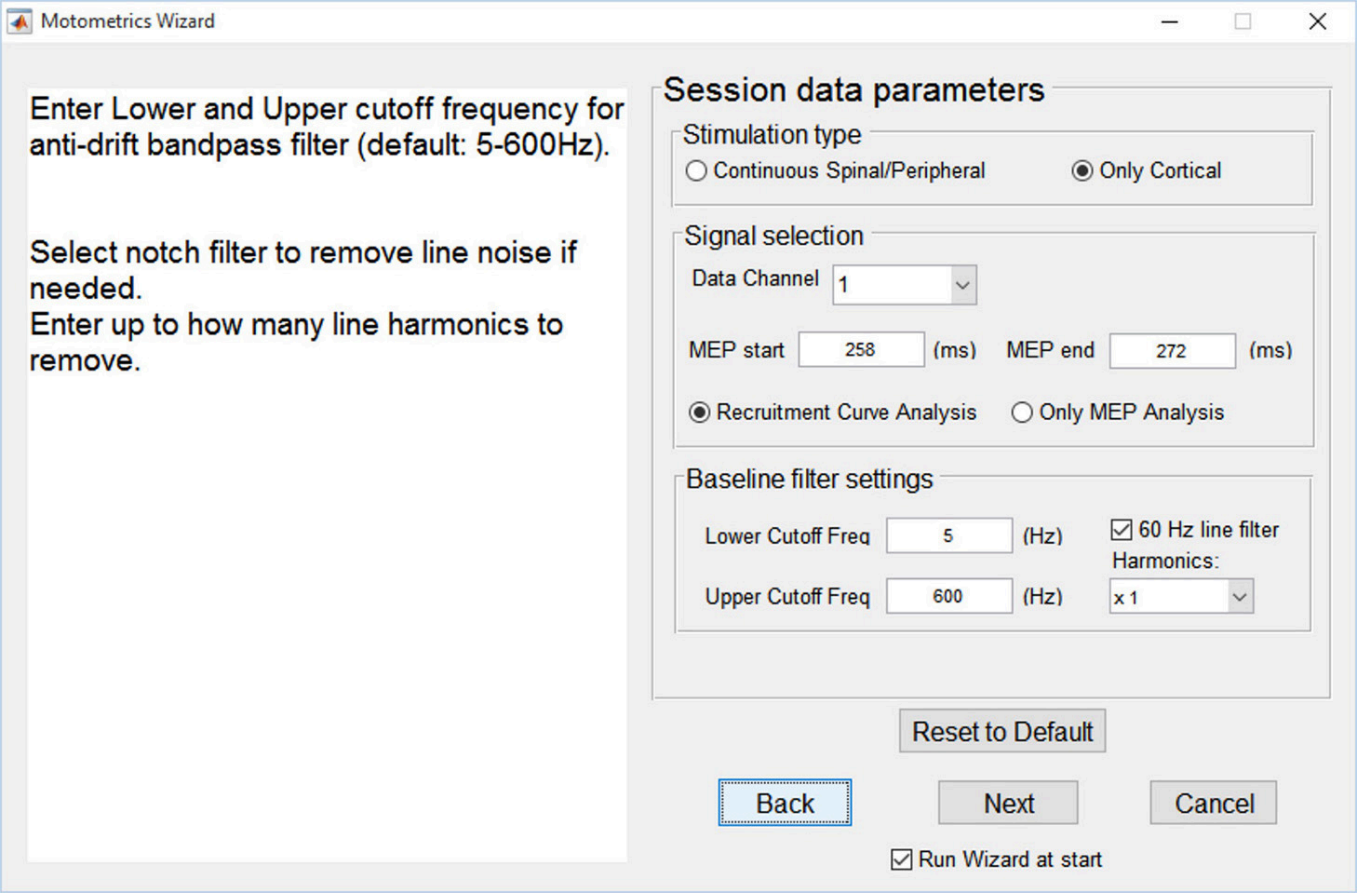
Motometrics wizard. The wizard guides the novice or unfamiliar user in selecting relevant parameter choices for configuring Motometrics. Each parameter is explained and acceptable ranges of values are suggested.

A parameter wizard was designed to serve as a simple GUI guide for configuring Motometrics parameters. This wizard is launched the first time Motometrics is run. It can also be launched at the click of a button from within Motometrics if the user desires. The wizard (Figure 5) assists the new user with step by step detailed descriptions to select MEP data for analysis, filter the data for artifact removal, control the curve fitting procedure, and set a threshold for detection of MEPs for the latency metric. The wizard also remembers the last modified set of parameters for use when Motometrics is run next, saving the user from re-entering them each time Motometrics is run. Additionally, the wizard enables a user to reset parameters to a default value if desired. The user may also load a sample recruitment curve session file in the wizard, to visualize the effects that their choice of parameters has on an MEP signal. While it is not mandatory to use the wizard to set parameters, it is recommended to do so for the new user. The more seasoned user may choose to skip the wizard and enter parameters directly into the main Motometrics window.

The primary goal of the parameter wizard is help neuroscientists without technical expertise to select appropriate values. Here, a non-technical user may use the default values for some or all of the parameters. The wizard also helps first time users configure Motometrics.

#### Data Transparency in Motometrics

For the advanced user Motometrics provides access to processed data corresponding to every step in the processing pipeline, i.e., screened raw data epochs, quantified MEP data, fitted recruitment curves and recruitment curve metric data. All of this data may be visualized directly using Motometrics as we show in the Results section. Optionally, the advanced user may save the data as structures in MATLAB data files and choose to run their own procedures on any of these data for verification, custom analyses or statistical interpretation. The quantified MEP data is saved as the following structure:


**Motometric_data: <**main structure name>
 |
 **File**: <name of master file>
 **Record(s):** <structure containing data and information about each experimental condition>
  |
  **File:** <name of record file>
  **Expt(s):** <structure holding MEP data for each experimental condition type>
   |
   **File:** <name of session file>
   **Start_msec:** <user specified start time of MEP>
   **End_msec:** <user specified end time of MEP>
   **Analysis_channel:** <user specified signal channel for selecting MEP data>
   **Filtered:** <0 or 1 indicating if signal was filtered or not>
   **Sampling_Freq:** <Sampling rate of MEP signal>
   **Stim_Range:** <vector of stimulation intensities>
   **Trials_per_stim:** <vector listing number of trials for each stimulation intensity>
   **Unprocessed_segmented_data:** <raw extracted MEPs before quantification>
   **Processed_mean_quantified_MEP:** <vector of mean across trials for each stim intensity>
   **Processed_quantified_MEP :** <cell array of vectors for quantified MEPs for each MEP signal across stim intensity and trials>
   **MEP_quantification_metric:** <String description of the MEP quantification type>
   **Curve_fit:** <structure containing fitted curves data>
    |
    **parameters:** <estimated parameters for fitting sigmoidal curve>
    **error:** <curve fitting error>
    **tolerance:** <User specified parameter for controlling goodness of fit vs. outlier resistance>
    **rec_curve_x:** <vector of interpolated values forming the x axis of the curve>
    **rec_curve_y:** <vector of estimated values forming the y axis of the curve>


The recruitment curve analysis data is saved in the following structure:


**Motometric_Analyzed_Data:** <main structure name>
 |
 **MetricType:** <cell array containing strings describing the type of recruitment curve metrics used>
 **Metric_CutOff:** <cell array containing %MEP or %Stim values corresponding to and depending on MetricType>
 **Rec_Metrics:** <structure array containing data for each recruitment curve metric type corresponding to MetricType>
  |
  **Bar_data:** <mean % values of the corresponding recruitment curve metric select. Mean is computed across multiple records (subjects)>
  **Bar_error:** <standard errors computed for the corresponding means>


The user may access saved data using these structures to perform any additional analyses they require. This can be done via custom scripts or from the MATLAB command window.

### Software Development and Architecture

**Figure 6:**
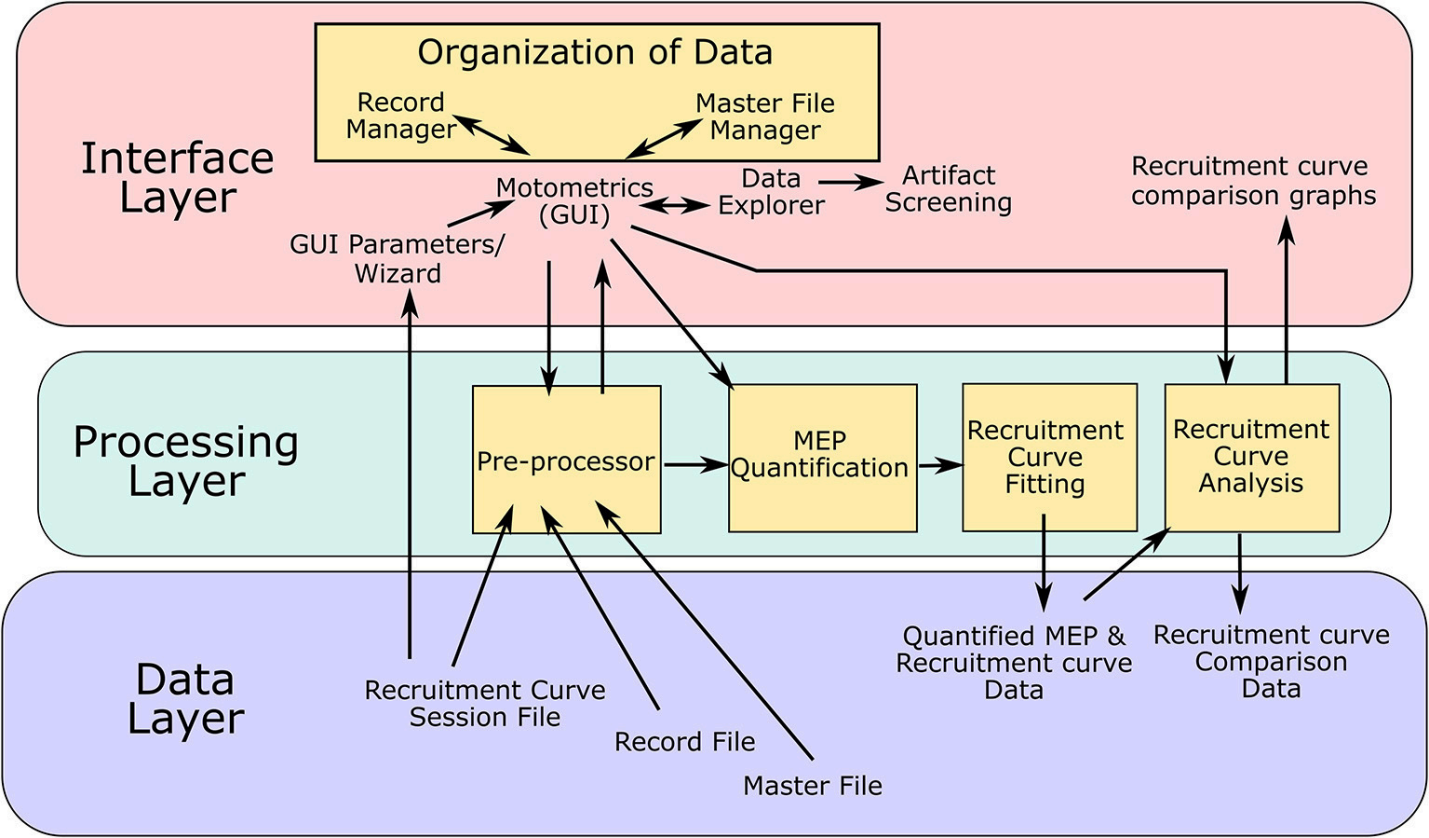
Software architecture**:** Motometrics uses a 3 layered software architecture model to achieve a modular design. The interface layer contains MATLAB function modules consisting of GUI windows to accept user input and provides graphical output to the user. The Data annotation modules are a part of this layer. The processing layer consists of the back end modules that extract and process MEP data to generate quantified MEPs and recruitment curves. The modules in yellow boxes correspond to the conceptual modules listed in Figure 2. The data layer represents the various data files and data structures that Motometrics reads and saves to for further analysis.

Motometrics was created using an iterative development model. Key features for the GUI and key modules for processing MEP and recruitment curve data were first identified and then through an iterative process, additional features, fixes, and enhancements were made based on testing by experimentalists. The software architecture that was used was the modular “3-tier layered model” as illustrated in Figure 6. Here, the key layers are the interface layer, the processing layer and the data layer. The arrows indicate the flow of information between the various modules and data.

The interface layer is the interactive front end of Motometrics that enables the user to specify various options, parameters, annotations, and selection of data. This layer accepts inputs but also provides graphical views of the data and analyses. It consists of the main Motometrics GUI, the parameter wizard, the core data organization module, and the data screening tool. This layer also holds the graphical output of the analyses chosen by the user. The processing layer is the backend of Motometrics. It consists of the data pre-processing module, the MEP quantification module, the recruitment curve fitting module, and the recruitment curve analysis module.

The final layer is the data layer. The data files and their data structures have been described in sections Data Organization and Annotation and Motometrics for the non-technical and advanced user. This layer holds both the input data that feeds into Motometrics (session files, record files, and master files) and output data (the quantified MEP and recruitment curve data as well as the recruitment curve comparative analysis data).

The interface and processing layer are highly modular in that changes to each module may be made without having to affect the other modules. The key modules that make up Motometrics are shown as orange boxes in Figure 6. These physical software modules directly correspond the conceptual modules shown in Figure 2.

### *In vivo* Experimental Setup for Demonstration Experiment

To demonstrate the utility of Motometrics, we performed experiments in 4 Sprague Dawley female rats. All protocols were approved by the Institutional Animal Care and Use Committee of Weill Cornell Medicine. MEPs were evoked by stimulating forelimb area of motor cortex with epidural screw electrodes and recording from the contralateral biceps muscle with implanted EMG wires as described in Sindhurakar et al. (2017). To modulate the MEP, low level (subthreshold) spinal cord stimulation was applied as described in Mishra et al. (2017). The paradigm was designed to augment motor cortex responses by spinal cord stimulation. Recruitment curves were created with no spinal cord stimulation (baseline condition) followed by recruitment curves with spinal cord stimulation, at a latency of 9, 10, and 11 ms from motor cortex stimulation. Multichannel EMG signals were continuously acquired at 5,000 Hz sampling rate, with a CED (CED Micro 1401, Cambridge Electronic Design Ltd, Cambridge, UK) data acquisition system and a recording software (Signal 5.08, CED Ltd). The signals were amplified at a gain of x1000 and bandpass filtered 1–1000 Hz with an integrated AC differential amplifier system (A-M Systems, Model 1700, Sequim, WA, USA). Motor cortex and spinal stimulation were performed using an isolated pulse stimulator (A-M Systems, Model 2100). The MEP files were saved as .mat files, compatible with MATLAB for Motometrics analysis.

## Results

In this section, we use an example dataset to illustrate the functions of Motometrics. We will step through the 5 core components (Figure 2) and present results at each step. The data used here was obtained from 4 rats after using the *in vivo* experimental setup described in Methods Section *in vivo Experimental Setup*. MEPs for recruitment curves were recorded under different conditions: Baseline (no spinal cord stimulation) and spinal cord stimulation applied 9, 10, or 11 ms after cortical stimulation. We have also successfully tested Motometrics on similarly structured MATLAB datafiles imported from three different systems: Cambridge Electronic Design, Tucker Davis Technologies, and AD Instruments. The sample code for converting these MATLAB data files to Motometrics compatible datafiles is provided along with this software under the folder “ConversionExampleScripts” in the main project directory.

### Annotating and Grouping Data

MEP recruitment curve session data was obtained from the system described in the experimental setup and the data in MATLAB files formatted for import into Motometrics, as described in Methods section *Structure of Recruitment Curve Session files*. An example recruitment curve session file and formatting script is included in the Motometrics repository.

#### Annotating MEP Data Files to Create Record Files

**Figure 7:**
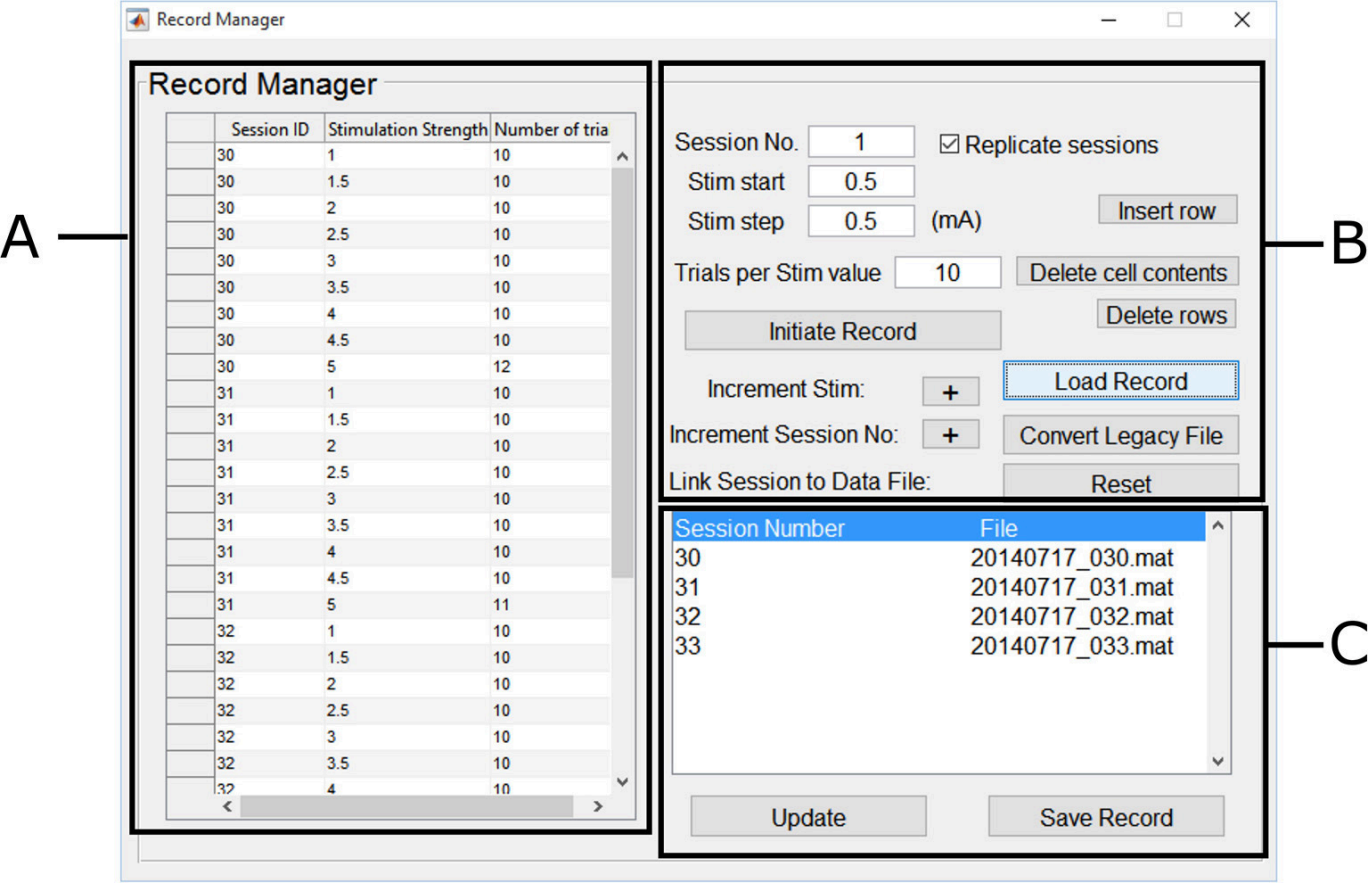
Motometrics record manager. The Record Manager enables one to annotate and group session data. **(A)** The table on the left, enables the user to input annotation information such as a unique session ID that corresponds to a specific experimental condition. This is generated automatically from the parameters on the right, but these can be changed within the table. **(B)** The controls on the right assist in the annotation process by identifying the session ID number and stimulation strength. Keyboard shortcuts can be used for faster annotation. **(C)** The list on the bottom right enables each session ID/experimental condition to be linked to the recruitment curve session file to create the record file. Previously created record files may also be loaded later for editing.

MEP session data files were converted to MATLAB data files, we annotated them using the Record Manager. We chose a unique ID for each experimental condition corresponding to a recruitment curve session, the range of stimulation intensities used, and the number of trials performed per stimulus intensity. The Motometrics Record Manager GUI is shown in Figure 7.

In the Record Manager, we began annotation using the forms and buttons shown in Figure 7B by specifying the first session ID (arbitrary, but must be unique). We also specified the first stimulus intensity used (0.5 mA), and the stimulation increment values (0.5 mA), and finally we specified how many trials were recorded for that intensity (10 trials per stimulation intensity, except for a few cases). We clicked on the *Initialize Record* button, followed by clicking on the *Increment Stim* button to add more stim values with the same number of trials within the same session ID. Annotation values that needed to be changed, were edited directly by typing in the table shown in Figure 7A. The buttons in Figure 7B also allow for deleting table cells and rows.

The process was repeated for more intensities until we were done describing the first recruitment curve session/experimental condition. We next clicked on the *increment session* button to indicate the beginning of a new experimental condition (recruitment curve session) and repeated the annotation process for the next recruitment curve session. To complete the creation of the record, we specified the recruitment curve session data files for each unique session ID. The GUI in Figure 7C automatically lists the unique session numbers under the *Session Number* column. The *File* column lists the file that has been linked to a particular session number. If no file has been linked, a “MissingFile” string placeholder is used instead. By double clicking on a session number in the list, a file browser dialog box was displayed, which enabled us to select the relevant MEP session data files that were linked to each session number. Finally clicking on *Save Record* created the record file. This process was repeated for recruitment curve session data across the 4 rats, where each record file corresponded to an individual rat. An experienced user was able to annotate and create a single record file in under a minute.

#### Grouping Records to Create Master Files

In order to compare recruitment curves across multiple rats, we grouped record files for all the rats into a single master file. This was done by clicking on the “Master File Manager” button, which brought up a window that enabled one to add or remove record files to a list.

### Data Selection and Pre-processing

After creating the record files and the master file, we next provided information to select the relevant data within each record. Specifically, a start (8 ms) and stop time (22 ms) was inputted to extract the MEP segment that occurred after stimulation. The corresponding channel was also specified to focus on the muscle of interest (biceps). Motometrics provides an optional band-pass filter option to correct for baseline drifting and large artifact removal (also configurable directly or through the parameter wizard); in this dataset, it was not used.

**Figure 8:**
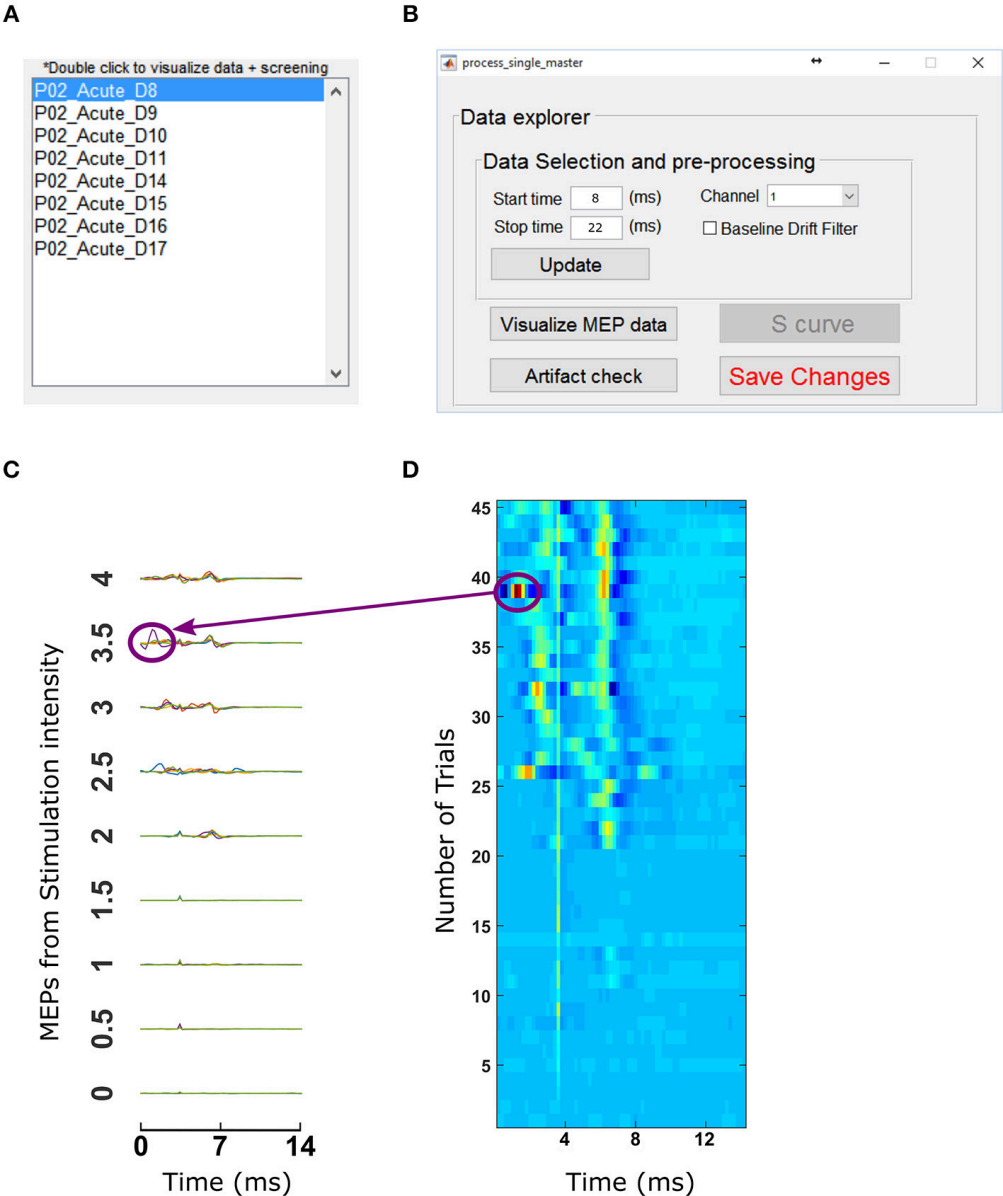
Data selection and visualization tools. **(A)** Motometrics provides a list of record files that can be processed with the data exploration tool by double clicking on any of them. **(B)** The data explorer tool enables the user to visualize MEP data or screen for artifacts. **(C)** The Visualization tool enables the user to examine MEP data that are extracted from the MEP session files specified in a record. **(D)** The artifact screening tool enables the user to obtain a graphical overview of all MEP data of a recruitment curve session in a single figure. The heat map representation makes it easy for the user to eyeball the data in order to spot inconsistencies or artifacts.

With the chosen data selection parameters entered, we then loaded the master file created earlier. The list of record files grouped under the master file was listed in the Motometrics GUI as shown in Figure 8A. Double clicking on any of the record files opened the data exploration tool, as shown in Figure 8B.

In order to inspect the MEP signals, we clicked on the *Visualize MEP data* button which then displayed MEP signals across all trials and stimulation intensities for each session/experimental condition in a record as shown in Figure 8C. Upon noticing a potential artifact (circled in Figure 8C), we then screened for outliers by clicking on the “Artifact check” button in the data explorer window. This brought up a window with heat maps of datasets as shown in Figure 8D. For Figures 8C,D the artifacts come from an example dataset with a single artifact. The dataset has 45 trials per recruitment curve session, with 9 stimulation intensities and each stimulation intensity having 5 trials. In the heat map, the blue end of the spectrum indicates low MEP values and red end of the spectrum indicated high MEP values. By observing discontinuous color ranges we were able to visually pick out an artifact. Clicking on this anomalous trial, allowed us to confirm that it was an artifact and delete it from the corresponding recruitment curve session file in our analysis. After this was done, clicking “*update*” and “*Save Changes*” on the data explorer window (Figure 8B) loads data without the trials marked for deletion.

### MEP Quantification and Generation of Recruitment Curves

**Figure 9:**
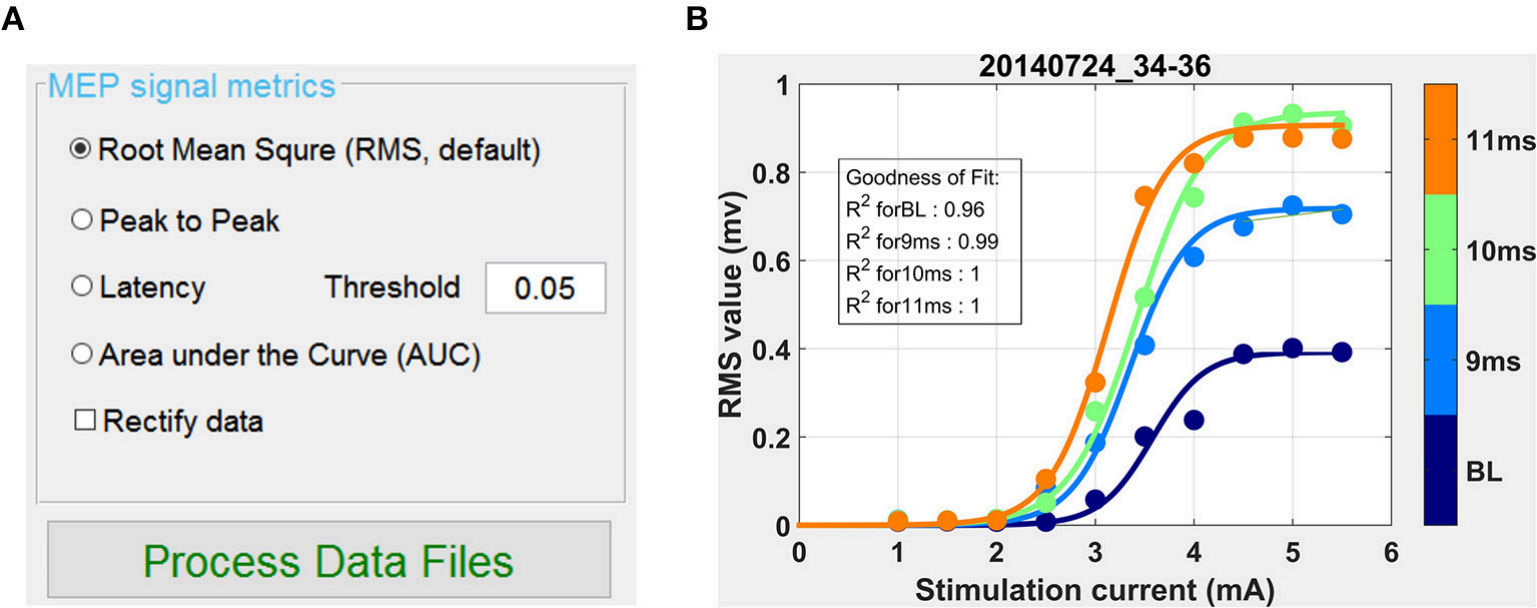
Quantifying MEPs and visualizing recruitment curves. **(A)** This GUI in the window of Motometrics window provides choices for different MEP metrics. **(B)** After the choice of MEP quantification metric is made, and the *Process Data Files* button is clicked, recruitment curves are generated.

Once data was annotated, grouped by experimental condition and screened for artifacts, Motometrics was next used to quantify the MEPs. This was done using the MEP metrics GUI shown in Figure 9A. We chose the root mean square quantification metric, selected a curve fit tolerance of 0.1, and clicked on the *Process Data Files* button to quantify MEPs. This process also automatically generated recruitment curves. On an Intel Core-i7 computer with 16 GB RAM, quantifying MEPs and fitting recruitment curves for 250 MB of data took 0.8 seconds in total. Generated recruitment curves (Figure 9B) could be immediately viewed by clicking on the “S curve” button (Figure 8B). Recruitment curves were generated for each MEP session/experimental condition and the goodness of fit was estimated using the R^2^ function (residual sum of squares). Additionally, when viewing the curves, the software indicates a warning if any of the curves appear to not saturate at maximum stimulation intensity; no warnings were produced for this data set. Extracted MEP data, quantified MEP data, and recruitment curve fits were all saved using the “*Save Quantified MEP Data*” button.

### Analysis and Comparison of Recruitment Curves

**Figure 10:**
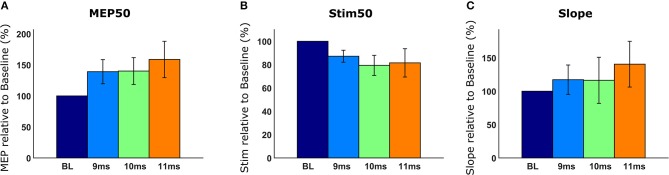
Analyzed recruitment curves. Recruitment curves are normalized to a baseline reference and displayed. **(A)** The MEP50 metric compares MEP magnitudes of recruitment curves given the stimulation intensity that produces 50% of maximum baseline MEP. **(B)** The Stim50 metric compares the amount of stimulation required for each recruitment curve to produce a MEP magnitude equal to the Baseline recruitment curve when 50% of maximum stimulation is used. **(C)** The Slope metric compares the rate of MEP increase with increasing stimulation intensity.

After quantifying MEPs and generating recruitment curves, we compared recruitment curves across conditions. We used the following recruitment curve metrics: MEP50, Stim50, and slope, which have been described in detail in section *Recruitment Curve Analysis*. Briefly, the MEP50 metric compared MEP magnitudes of all recruitment curves for a corresponding stimulation intensity that produces 50% of maximum baseline MEP. The Stim50 metric compared the required stimulus intensities across all recruitment curves, for producing an MEP magnitude elicited by 50% of maximum baseline stimulus. When the desired recruitment curve metrics were selected and the “Generate Analyses” button was clicked, we obtained graphical results of our requested analyses as shown in Figure 10. In Figure 10A, we observed that when the spinal cord is stimulated 11 ms after brain stimulation, we got the greatest enhancement, indicating a higher excitability of the motor system. In Figure 10B, we compared the Stim50 metric across recruitment curves. The lower the Stim50 value for a recruitment curve, the more responsive the motor system is.; 10 and 11 ms had the smallest Stim50 values corresponding to more excitable states of the motor system. Additionally, the rate of increase in MEP with stimulation, as measured by the slope, was highest for the case of spinal cord being stimulated with a 11 ms latency (Figure 10C). The comparative data provided by Motometrics after applying recruitment curve metrics, was saved, using the “*Save Analysis Data*” button for further statistical analysis.

## Discussion

Motometrics fills many of the gaps in the current state of MEP quantification and recruitment curve analysis that were identified in the Introduction. First, Motometrics simplified the process of analyzing MEPs and for fitting and analyzing recruitment curves. Motometrics was fast, enabling the experimentalist to perform near-real-time assays of generated recruitment curves during experimentation. There are currently various data file formats available from the different electrophysiology systems. For Motometrics to work with these files, it is required to convert them to a standard format as described in section Structure of Recruitment-Curve Session Files. We have provided sample data conversion scripts for two popular electrophysiology systems. These sample scripts can be edited as required to work with data from other electrophysiology system. Finally, Motometrics provided high levels of access to parameters and data for a range of users, from novice to advanced.

In our results, Motometrics was tested by a neuroscientist with minimal signal processing and MATLAB background who was experienced in using Motometrics. MEP data during experiments were quickly annotated as record files under a minute. These record files were grouped as a master file which was processed for MEP quantification and recruitment curve fitting. This part of the process took less than a second for 250 MB of MEP data. The neuroscientist was also able to easily edit previously created record files as needed. We believe that this serves as evidence that Motometrics was sufficiently fast to enable near real time analysis independent of the level of the user's technical expertise. Additionally, Motometrics was tested on example data imported from Cambridge Electronic Design, Tucker Davis Technologies, and AD Instruments, indicating its capability in handling data from multiple electrophysiology systems.

Motometrics has features necessary for quantifying MEPs, fitting recruitment curves, and analyzing fitted curves. The range of analysis features provided by Motometrics gives the user many options in analyzing and interpreting his or her data. This saves users time by removing their focus from implementing analytical methods, and instead enable them to focus on the relevant experiments and outcomes. Since Motometrics is hardware independent, it can also facilitate collaboration and assimilation of data across labs regardless of which electrophysiological system is used.

Motometrics can serve the analytical needs for a wide range of researchers. Current users are those who either want to comparatively analyze quantified MEP values directly, or those who wish to generate and compare recruitment curves across different experimental conditions. The access to data and parameters in Motometrics enables a user to extract data at different points in the analysis pipeline and run their own custom analyses. For example, while previous literature has strongly supported the sigmoidal curve for modeling recruitment curves (Fuglevand et al., 1993; Klimstra and Zehr, 2008), some users may prefer other custom functions to generate their recruitment curves. With the data and parameter accessibility features, users can not only control the degree of sigmoid curve fit to their data but also the ability to extract quantified MEP data and run their custom curve fitting and subsequent analyses. Also, the modular nature of Motometrics, can enable the user to add their custom analyses permanently to the processing pipeline.

Despite the salient features listed above, we acknowledge that Motometrics has a few limitations. Firstly, rare changes in MATLAB coding interfaces across different versions can break the function of previously coded programs like Motometrics. This issue may be avoided by using MATLAB 2015 or later. We will also maintain the code to be compatible with future versions. The second limitation, as stated previously, is that we assume a sigmoidal recruitment curve for fitting. Users may prefer the ability to fit data to custom curve equations. Based on user feedback, this can be potentially implemented in the future. Finally, with newer electrophysiology systems, it is possible to obtain stimulation information directly. This information along with a user provided standard experimental template would mean that annotation may possibly be automated. Additionally, parameters such as MEP start and stop times may be directly inferred instead of being entered manually. Automating annotation and signal selection are future goals for Motometrics.

As an open source tool, Motometrics is accessible to experimentalists for analyzing their MEP data and for fitting recruitment curves and analyzing these curves. In addition, Motometrics can analyze MEP data from nerve as well as muscle. For example, an unaffiliated lab tested the use of Motometrics for analyzing electroneurography (ENG) signals in response to stimulation. Motometrics was able to provide quick quantification and generation of the relevant recruitment curves (not shown here) from ENG, similar to what was shown for EMG. Furthermore, with its modular design Motometrics enables experienced MATLAB programmers to add custom functionality as needed. In conclusion, Motometrics enabled advanced signal processing and near real time analysis of recruitment curves, and its modular nature and simplicity can also enable it to be extended for new analytical paradigms.

## Ethics Statement

This study was carried out in accordance with the recommendations of National Institutes of Health guidelines, committee of Weill Cornell Medicine. The protocol was approved by the committee of Weill Cornell Medicine.

## Author Contributions

JC and DG conceptualized the problem. DG designed the initial approach with assistance from NH. SR conceptualized, designed Motometrics, and expanded on the work by DG. AP and AM extensively tested Motometrics in experimental settings and provided feedback and corresponding analytical results. SR wrote the paper with significant input from JC and DG.

### Conflict of Interest Statement

The authors declare that the research was conducted in the absence of any commercial or financial relationships that could be construed as a potential conflict of interest.
